# Study on prognosis of acutely ruptured intracranial aneurysms (SPARTA): a protocol for a multicentre prospective cohort study

**DOI:** 10.1186/s12883-024-03567-6

**Published:** 2024-02-17

**Authors:** Alexander L. Hamming, Jeroen T.J.M. van Dijck, Tjitske Visser, Martine Baarse, Dagmar Verbaan, Hanna Schenck, Roel H.L. Haeren, Rahman Fakhry, Ruben Dammers, René Aquarius, Jeroen H.D. Boogaarts, Wilco C. Peul, Wouter A. Moojen

**Affiliations:** 1 University Neurosurgical Centre Holland, Leiden University Medical Centre, Haaglanden Medical Centre and Haga Hospital, Leiden and The Hague, Lijnbaanweg 32, The Hague, 2512 VA The Netherlands; 2 Department of Neurosurgery, Amsterdam University Medical Centre, Amsterdam, The Netherlands; 3 Department of Neurosurgery, Maastricht University Medical Centre, Maastricht, The Netherlands; 4 Department of Neurosurgery, Erasmus MC Stroke Centre, Erasmus Medical Centre, Rotterdam, The Netherlands; 5 Department of Neurosurgery, Radboud University Medical Centre, Nijmegen, The Netherlands

**Keywords:** Aneurysmal subarachnoid haemorrhage, Ruptured intracranial aneurysm, Endovascular treatment, Clip-reconstruction, Neurosurgery, Long-term outcome, Cost-effectiveness, Comparative effectiveness

## Abstract

**Background:**

Ruptured intracranial aneurysms resulting in subarachnoid haemorrhage can be treated by open surgical or endovascular treatment. Despite multiple previous studies, uncertainties on the optimal treatment practice still exists. The resulting treatment variation may result in a variable, potentially worse, patient outcome. To better inform future treatment strategies, this study aims to identify the effectiveness of different treatment strategies in patients with ruptured intracranial aneurysms by investigating long-term functional outcome, complications and cost-effectiveness. An explorative analysis of the diagnostic and prognostic value of radiological imaging will also be performed.

**Methods:**

This multi-centre observational prospective cohort study will have a follow-up of 10 years. A total of 880 adult patients with a subarachnoid haemorrhage caused by a ruptured intracranial aneurysm will be included. Calculation of sample size (*N* = 880) was performed to show non-inferiority of clip-reconstruction compared to endovascular treatment on 1 year outcome, assessed by using the ordinal modified Rankin Scale. The primary endpoint is the modified Rankin Scale score and mortality at 1 year after the initial subarachnoid haemorrhage. Patients will receive ‘non-experimental’ regular care during their hospital stay. For this study, health questionnaires and functional outcome will be assessed at baseline, before discharge and at follow-up visits.

**Discussion:**

Despite the major healthcare and societal burden, the optimal treatment strategy for patients with subarachnoid haemorrhage caused by ruptured intracranial aneurysms is yet to be determined. Findings of this comparative effectiveness study, in which in-between centre variation in practice and patient outcome are investigated, will provide evidence on the effectiveness of treatment strategies, hopefully contributing to future high value treatment standardisation.

**Trial registration number:**

NCT05851989

**Date of registration:**

May 10th, 2023

## Background

Spontaneous subarachnoid haemorrhage (SAH) is a life-threatening event, most frequently (around 85% of cases) caused by the rupture of an intracranial aneurysm (IA). The prevalence of unruptured IAs in the general population is estimated to be 1.8% at a mean age of 63.3 years and women are affected more often than men [1, 2]. In the Netherlands, the incidence of aneurysmal SAH (aSAH) is estimated to be 8–9 per 100.000 per year and the average annual incidence from 2014 to 2017 according to the Dutch Quality Registry for Neuro Surgery (QRNS) is 1014 cases [3–5]. It is generally believed that 8–10% of the patients die before hospital arrival after an SAH [6]. Mortality after aneurysmal SAH for Europe excluding northern Sweden and Finland is thought to be 44.4% [3, 4, 7]. The mortality rate, however, seems to be declining due to improved prehospital and hospital care [3, 7]. When considering functional outcome, it is assumed that approximately a third of all patients with an aSAH has a “good functional outcome”, i.e. mRS ≤ 2 [8].

An aSAH is a complex pathology requiring close monitoring in the first weeks following admission. The primary treatment for ruptured IAs is performed to prevent the high-risk occurrence of early rebleeding by exclusion of the aneurysm from the circulation. Furthermore, delayed cerebral ischemia (DCI) and hydrocephalus are both common and dangerous consequences of SAH, often prompting specific medical and/or surgical therapy [8]. Systemic complications including cardiac instability, hyponatremia and other metabolic dysregulation like hyperglycaemia can negatively affect patient outcome and require monitoring and treatment [9–11].

Nowadays, the most applied primary treatment modality of the ruptured aneurysm is endovascular treatment, which has replaced craniotomy with microsurgical clip-reconstruction in the majority of cases [12]. The best but conflicting evidence for this treatment strategy comes from the results of two randomized controlled trials: the Barrow Ruptured Aneurysm Trial (BRAT) and the International Subarachnoid Aneurysm Trial (ISAT) [13, 14]. The outcomes from the ISAT trial suggest that survival is slightly but significantly increased in patients treated by endovascular treatment compared to patients treated with microsurgical clip-reconstruction [13, 15, 16]. Initial results from the BRAT suggested superior functional outcome for patients treated with endovascular treatment, but these did not remain significant with longer follow-up [14, 17–19].

Results from the ISAT and BRAT demonstrate that the optimal evidence-based practice for treatment of acutely ruptured IAs has yet to be determined. The promising endovascular treatment, introduced two decades ago, seems to have a higher incidence of rebleeding and need for retreatment, necessitating intensive outpatient radiological follow-up and therefore keeps a large proportion of treated patients unsure about their future. This uncertainty is very debilitating as the risk of a recurring bleeding incident has an impact on the patient, their family and society (loss of work).

The choice of endovascular treatment or clip-reconstruction is primarily decided by the treating physician based on the aneurysm location, morphology and local and physician-bound treatment paradigms that take the clinical condition of the patient into account. Hospitals may have different treating algorithms for similar patients including endovascular techniques and microsurgical techniques. Aside from the treatment modality, even deciding whether to treat ruptured IAs immediately after presentation, to postpone treatment or to choose not to treat the causative aneurysm, especially in cases with severely affected patients (grades IV or V according to the World Federation of Neurosurgical Societies (WFNS)-grading system) is variable.

### Study aim

The aim of this prospective observational cohort study is to identify the most effective treatment strategy for the best outcome in patients with a subarachnoid haemorrhage caused by a ruptured intracranial aneurysm.

## Methods

### Primary objective and research question

The primary objective is to determine the effect of clip-reconstruction versus endovascular treatment on the functional outcome at 1 year in patients presenting with an SAH due to a ruptured IA. We hypothesize that the functional outcome of clip-reconstruction is non-inferior to endovascular treatment at 1 year after treatment, but that inherent treatment differences might become apparent beyond this horizon.

### Secondary objectives

Secondary objectives include the effect of clip-reconstruction versus endovascular treatment on the functional outcome and mortality of patients with ruptured AIs at 6 months, 2, 5 and 10 years after ictus. Besides functional outcome, this study will examine the effect of clip-reconstruction versus endovascular treatment on neuropsychological outcome, quality of life and the need for rehabilitation. Furthermore, timing of treatment and differences in outcome between primary treatment modalities, including advanced endovascular treatment techniques, will be examined. In addition, secondary treatments (for hydrocephalus or DCI) and the effect on the named outcome parameters will be studied. Also, computed tomography (CT)- and angiographic imaging data will be collected to evaluate prognostic factors for surgical outcome, functional outcome and risk of complications after primary or secondary treatment. Lastly, an estimate of the costs associated with primary treatment modalities, supportive care measures and care settings, different treatments for complications, long-term follow-up imaging and different rehabilitation strategies will be established. The role of different referral area, caseload and number of care providers when considering functional outcome after aSAH will be determined.

### Study design

#### Study design

Longitudinal multicentre prospective observational cohort study.

During the study period, all SAH patients presenting at the study sites will be screened for inclusion eligibility. Following informed consent, eligible patients will be included as participants in the study. Treatment decisions and treatment methods (e.g. neurosurgical clip-reconstruction or endovascular treatment for primary treatment of the aneurysm) will not be influenced by inclusion in the study. Standard care tailored to the patient needs is provided, as is customary in the study site by the local treating team. Patient characteristics and initial treatment considerations will be collected for participants as baseline information.

Follow-up of the subjects is according the Dutch national quality regulations during the initial 6 months after aSAH ictus, and is tailored to the specific needs of the patient. At these regular follow-up visits, study questionnaires are completed. After regular follow-up visits, patients included in the study will have at least four more visits, at respectively 1, 2, 5 and 10 years after aSAH ictus. Additional visits may be necessary for patient-specific reasons and should be documented, but are not required by the study. During these study visits, patients will complete questionnaires and the local treating team will report on the treatment and clinical findings.

The primary outcome measurement will be evaluated after the 1-year follow-up of the last included patient. The study will be completed after 10 years of follow-up.

#### Study sites

To answer the research questions 5 centres from The Netherlands will include subjects.

Participating centres:

- University Neurosurgical Centre Holland Leiden – The Hague (UNCH), including the Leiden University Medical Centre (LUMC) in Leiden, HAGA hospital in The Hague and Haaglanden Medical Centre (HMC) in The Hague.
- Radboud University Medical Centre (Radboud UMC).
- Academic Medical Centre Amsterdam (AMC).
- Maastricht University Medical Centre (MUMC).
- Erasmus Medical Centre (EMC).

All of these study sites will collect data from their own patients in an electronic database and centralised analysis will be performed from the main study location in Leiden – The Hague.

A steering committee will be established to include local investigators in the management aspects surrounding the study, including publication policy and for the sake of transparency.

#### Sample size / sample size calculation

Calculation of sample size was performed to show non-inferiority of clip-reconstruction compared to endovascular treatment on 1-year outcome, as assessed using the ordinal modified Rankin Scale. The software of Sealed Envelope was used for calculation of the sample size [20]. We assume that the rate of poor outcome (mRS < 2) after clip-reconstruction and endovascular treatment is equal (31%) based on the uncertainties in previous literature regarding selection of patients and biases [13–19]. The non-inferiority margin is set at 7% difference. This is based on the largest previous clipping versus coiling trial where an absolute risk reduction of death and dependency of 7% was found in coiling compared to clipping [13]. Alpha is set to 0.05 and beta to 0.8. Using these assumptions, we require a sample size of 1100 patients. Since analysing the full-modified Rankin Scale instead of dichotomizing it decreases the required sample size by approximately 20%, we therefore require 880 patients [21, 22].

#### Number of subjects

We will include 880 subjects.

#### Study duration

Based on the number of SAH patients that are admitted at the various study sites, we expect that inclusion of 880 patients will take around four to five years. Recruitment will start on July 2021 and is expected to finish on July 2026. Patients will have a maximum follow-up of 10 years, resulting in a maximum of 14 years data collection. Inclusion of patients stops when the targeted number of subjects is reached.

In the event of slower inclusion, the recruitment period may be extended.

### Study population

#### Population (base)

All patients that present primarily or after acute referral (in-patient to in-patient) to the participating centres, with a spontaneous subarachnoid haemorrhage due to a ruptured IA will be screened for eligibility for this study by the treating physicians or local research nurses.

#### Inclusion criteria

In order to be eligible to participate in this study, a subject must meet all the following criteria:

- Confirmed diagnosis of SAH on CT-scan or lumbar puncture (in the presence of a negative CT-scan).
- IA related SAH as confirmed with radiological imaging.
- Age 18 years or older at presentation.
- Written informed consent.

Written informed consent for participation in the study will be obtained from the patient or the legal representative during the admission period by the local treating team.

#### Exclusion criteria

A potential subject who meets any of the following criteria on presentation to one of the participating centres or later during the clinical course will be excluded from participation in this study:

- SAH deemed most likely of ’perimesencephalic’ origin after consideration of history, clinical examination and radiological findings (including angiographic imaging).
- SAH deemed most likely of post-traumatic origin after consideration of history, clinical examination and radiological findings (including angiographic imaging).
- Diagnosis of intracerebral arteriovenous malformations or dural arteriovenous fistula.
- No diagnosis of IA at 6 months after onset of symptoms.

Participant does not master the Dutch language sufficiently.

### Treatment of subjects

The treatment protocol for the patients included in the study is not specified by the study protocol, as the study is strictly observational with current practice care.

### Study parameters/endpoints

#### Main study parameter/endpoint

The primary endpoint in this study is the score on the mRS at 1 year after onset of symptoms.

This will be collected through a case report form filled in by the local treating team at the 6 month and 1-year interval after onset of symptoms. The mRS will be obtained through a structured interview, either by phone or at a face-to-face assessment [20]. Additionally, mortality before this point will be documented by the local treating team.

#### Secondary study parameters/endpoints

To summarize some of the most used outcome parameters we will use the following terms:

‘Functional outcome’-data set:

- mRS at 6 months, 1, 2, 5 and 10 years after onset of symptoms, as measured by completion of case report forms by the local treating teams.
- Barthel index at 6 months, 1, 2, 5 and 10 years after onset of symptoms, as measured by completion of case report forms by the local treating teams.

‘Cognitive outcome’-data set, meaning general cognitive functioning and mood/fatigue:

- Modified Telephone Interview for Cognitive Status (TICS-M) at 6 months, 1, 2, 5 and 10 years after onset as measured by completion of case report forms by the local treating teams.
- Hospital Anxiety and Depression Scale (HADS) at 6 months, 1, 2, 5 and 10 years after onset of symptoms by completion of questionnaires by the subjects.

‘Quality of life and Costs’-data set:

- The 5-level EQ-5D (EQ-5D-5 L) at 6 months, 1, 2, 5 and 10 years after onset of symptoms, as measured by completion of questionnaires by the subjects.
- Estimation of societal costs by completion of a custom healthcare consumption and loss of productivity (paid and unpaid) questionnaire at 3 months, 6 months, 1 year, 2 years, 5 years and 10 years completed by the subjects.

#### Primary treatment

- Case report form (CRF) on type of primary treatment, timing of treatment, reasoning for choice of treatment, per procedural complications, used material and time.
- CRF on the occurrence of rebleeding and retreatment, reasoning for choice of retreatment, modality of retreatment, timing of retreatment, used material and time.
- CRF on death of subject, timing of death, suspected cause of death.
- Functional outcome data set (see above).
- Cognitive outcome data set (see above).
- Quality of life data set (see above).

#### Secondary treatment

- CRF on the occurrence of DCI and its treatment, timing of treatment, reasoning for choice of treatment, per procedural complications, used material and time.
- CRF on the occurrence of hydrocephalus and its treatment, timing of treatment.
- CRF on the admission of patient to ICU or medium care unit, reasoning for choice of ward.
- CRF on death of subject, timing of death, suspected cause of death.
- Functional outcome data set (see above).
- Cognitive outcome data set (see above).
- Quality of life data set (see above).

#### Rehabilitation

- CRF on the rehabilitation choice for patients, reasoning for choice, timing of rehabilitation and intensity of programme.
- Functional outcome data set (see above).
- Cognitive outcome data set (see above).
- Quality of life data set (see above).

#### Follow-up and imaging

- CRF on the follow-up schedule, reasoning for the schedule, used time and imaging requested.
- CRF on aneurysm recanalization and recurrence, describing aneurysm morphology, changes, clinical course.
- Initial CT and (CT-)angiography digital images and centralized analysis by blinded radiology panel.

#### Cost-effectiveness and organization of care

- All CRF’s mentioned above, except the CRF’s on recanalization and death.
- Hospital specific referral area, caseload, number of care providers.
- Functional outcome data set (see above).
- Cognitive outcome data set (see above).
- Quality of life data set (see above).

#### Baseline information

- Baseline patient information CRF on common data elements.
- All CRF’s above.
- Functional outcome data set (see above).
- Cognitive outcome data set (see above).
- Quality of life data set (see above).

### Study procedures

Study procedures for inclusion in the study are the same in all study sites. The local treating physicians will obtain informed consent and treat patients according to local hospital protocol or their own clinical insight. The data collected in this study will be collected without interfering with treatment of the patient and is strictly observational, supplemented by questionnaires.

The procedure of informed consent for participation in the study will be discussed in the section ‘Recruitment and consent’.

Baseline information, treatment information, outcomes and imaging data will be collected according to specified time points. The follow-up of all patients will be 10 years after ictus. The local treating physician and other appropriate treatment providers will fill out the questionnaires and perform the necessary tests. The data will be entered into the database directly when possible. A research-assistant will enter the data into the database at a later point if this is not possible at some locations.

All measurements will be performed by qualified physicians or research nurses that have received training to perform the measurements. The measurements will take place during follow-up appointments, by phone, by (e-)mail and will be registered in Castor, an online data capture environment. In cases where patients have difficulty completing the form, legal representatives or family or close caregivers can assist them in completing the form. Patients that have a lowered level of consciousness will be evaluated by physicians or research nurses trained to handle these specific patients. After assignment of a patient-specific study code, the data will be entered into a centralised electronic database in the different study sites by treating physicians or study nurses, according to local organisation. The Masterfile containing the link between the identifiable patient data and the study code will be kept in the local study site. Process related variables and associated costs will be acquired from participating centres.

After collection of this data in the centralised database, analysis will be primarily performed by the sponsor and the coordinating researchers. Local investigators may use the data from their centre for separate analyses after consensus in the steering committee.

### Timing of data acquisition

#### On baseline/admission

- Informed consent form
- Baseline information CRF.
- Initial CT and (CT-)angiography digital images.
- CRF on admission.

#### Primary treatment

- CRF on primary treatment.
- If rebleeding or retreatment, CRF on rebleeding and retreatment.

#### Secondary treatments

- CRF on patient mobilisation and inpatient rehabilitation.
- If hydrocephalus or DCI, CRF on hydrocephalus or DCI.

#### Hospital discharge

- Deferred informed consent form if not yet consented.
- If death, CRF on death.

#### Scheduled follow-up visits

- CRF on follow-up schedule.
- If rehabilitation referral, fill CRF on rehabilitation.
- If recanalization/retreatment, fill CRF on recanalization/retreatment.
- If death, fill CRF on death.
- At 3 months: costs questionnaire.
- At 6 months: mRS, TICS-m, Hospital Anxiety and Depression Scale (HADS), EQ-5D, Barthel ADL index, costs questionnaire.
- At 1 year: mRS, TICS-m, HADS, EQ-5D, Barthel index, costs questionnaire.
- At 2 year: mRS, TICS-m, HADS, EQ-5D, Barthel index, costs questionnaire.
- At 5 year: mRS, TICS-m, HADS, EQ-5D, Barthel index, costs questionnaire.
- At 10 year: mRS, TICS-m HADS, EQ-5D, Barthel index, costs questionnaire.

### Withdrawal of individual subjects

Subjects can leave the study at any time for any reason if they wish to do so without any further consequences. The patient is then requested, however not required, to complete a questionnaire on withdrawal from the study.

#### Replacement of individual subjects after withdrawal

Subjects who are withdrawn from the study will not be replaced.

#### Follow-up of subjects withdrawn from treatment

General category of reason for withdrawal should be completed on the CRF. After this, there is no study follow up of patients after withdrawal. Regular clinical follow up is maintained as decided by the local treating team.

### Safety reporting

Aneurysmal SAH is a life-altering event that can lead to temporary or permanent discomfort or injury and even death. This is unfortunately inherent to the disease and this study will not interfere with the clinical course of this disease by any intervention. As the study is observational in nature comparing different forms of accepted practice, it is unlikely that any events will occur that are caused by participation in the study.

Information on the course of the disease in the study subjects will be gathered in the study. As such, CRFs require local investigators to report complications of the SAH. We will not report AEs, SAEs or SUSARs to the METC but include them in the CRF.

No committee regarding the safety of patients will be established, as the safety is not at risk in the observational design of the study. A committee evaluating the correct performance of the study will be convened because of the long-term run of the study and multicentre design, a so-called data safety monitoring board. The charter is attached.

### Statistical analysis

#### Primary study parameter

To examine effectiveness of the interventions, proportional odds logistic regression models with ordinal mRS as outcome variable will be used. This method increases statistical power when compared to reporting on a dichotomized ordinal scale. We will report the odds ratios per cut-off and an average (common) odds ratio to give insight in fulfilment of the requisites of the proportional odds model [23].

To account for the risk of confounding by indication in the observational design, correction will be performed based on the aneurysm location, morphology and current clinical patient status. Strongest predictors of outcome from literature (age, history of hypertension, WFNS grading on admission, CT blood clot burden, aneurysm location and size, treatment variables, cerebral ischemia [24–27]) will be added as covariates. Analysis will be performed in R and in the Statistical Package for the Social Sciences (SPSS) version 21. A *p*-value < 0.05 will be considered statistically significant.

#### Secondary study parameter(s)

As a secondary study parameter, we will analyse the between-hospital differences to correct for confounding. Conventional methods are likely unable to account for the (unmeasured) confounding in SAH, like in traumatic brain injury [28]. Therefore, the main analyses will use the between-hospital variation in treatment for determining effectiveness by comparing regional treatment strategies. This is an instrumental variable approach [28]. The instrument is treatment preference and is determined as the amount of either coiled or clipped patients of total amount of treated patients. The proportion (percentage) exposed to the intervention in each hospital (the instrument) is entered as an independent variable to the analyses. The unmeasured and measured confounding at the hospital level, for example hospitals that perform more surgery also more often perform other treatments, is overcome with a multilevel model [29]. In this model, the random intercept should capture the measured and unmeasured confounders at hospital level, resulting in unbiased treatment effect estimates. The random intercept for each hospital represents the unexplained hospital effect (beyond all factors included in the model, including the instrument treatment preference).

In sensitivity analyses, the instrument validity will be further explored by quantifying a priori collected data and the results of provider profiling of SPARTA and comparing these to the posthoc derived relative proportion exposed to the intervention per hospital.

Moreover, as an alternative to the instrumental variable approach of the primary analyses, the instrument is modelled as a categorical variable. Specifically, the hospitals are divided into halves, tertiles and/or quartiles based on their preference for the intervention.

As secondary analyses, conventional ordinal regression analyses are planned with actual treatment as binary treatment variable and mRS as outcome variable. For aneurysm treatment effectiveness, confounding will be controlled for by adding known predictors for outcome as covariates in the model.

A similar strategy will be utilized to analyse the effectiveness of clip-reconstruction versus endovascular treatment on longer time intervals, the effectiveness of flow diversion and Woven EndoBridge®-devices, the effectiveness of secondary treatment modalities, timing of mobilisation and different rehabilitation programmes on the functional outcome using mRS and proportional odds regression modelling.

HADS for anxiety and depression will be analysed in a dichotomized way because of the uncertainty of abnormality in the borderline categories. The total score will be analysed using a proportional odds logistic regression model as described above. Different timing of treatment will be analysed both as a categorical variable (within 6 h, within 24 h, within 72 h, within 1 week, within 2 weeks, first month) and as a continuous variable.

Multivariate regression analysis and stepwise univariate analyses will be used for correlation of the imaging, patient specific and treatment data and outcome measurements, including occurrence of complications. Proportional odds regression modelling will be used for the analysis of recanalization and rebleeding using the reported morphological changes and occurrence of rebleeding.

Cost-effectiveness and cost-utility analysis will be performed using mRS at one year and QALY respectively. Effectiveness in QALYs, costs and cost-effectiveness ratios will be calculated. The analysis will use direct and indirect healthcare costs from both self-reported and CRF provided data, aspiring an extensive list of the costs in and out of hospital and productivity losses. Sensitivity analyses will be performed to validate the results [30, 31].

## Discussion

Spontaneous subarachnoid haemorrhage (SAH) caused by the rupture of an IA is a life-threatening event with high mortality rates [3, 4, 6]. Although mortality rates seem to be improving due to improved prehospital and in-hospital care [3, 6], only a third of all patients achieve a so called good outcome (mRS 0–2) [7]. Despite the major healthcare and societal burden, the optimal treatment strategy for these patients is yet to be determined. The overall objective of this study is to investigate the effectiveness of existing treatment strategies for patients with SAH caused by a ruptured IA in the Netherlands.

This study will evaluate the effect of clip-reconstruction versus endovascular treatment on functional and cognitive outcome, quality of life and cost-effectiveness at 1, 2, 5, and 10 years after onset of symptoms. As described earlier in this protocol, these essential pieces of information are largely unknown. Therefore, this study is a necessary addition to literature on the treatment of aSAH. The latter because the assessment of long-term treatment effectiveness is not possible due to insufficient data and because scientific analysis of these data is not permitted. This study also aims to improve insight into the full chain of care for these patients going through hospitals, rehabilitation centres, nursing homes or other outpatient settings. By employing comparative effectiveness analyses, in which in-between centre variation in clinical practice and patient outcome are investigated, the influence of treatment strategies on patient outcome will be investigated.

The longitudinal multicentre prospective observational cohort study design, instead of a randomized controlled trial (RCT), was selected to provide a more pragmatic and cost-effective assessment of treatment strategy effectiveness. Longitudinal observational studies are more feasible for long-term follow up of clinical outcomes and for investigating a relatively variable study population. This is more pragmatic and improves generalisability of study results. Investigating treatment- and cost-effectiveness through an RCT is complicated by the heterogeneity of aSAH patients, the sample size requirements, already established treatment strategies, the often life-threatening situation, and inadequate research budgets. The feasibility of this large-scale observational study is likely to be good due to its observational pragmatic design and due to the extensive experience obtained from participating in the CENTRE-TBI study [32]. The collection of extensive primary and secondary outcome measures is another strength of this study. Nonetheless, data collection could be challenging by its extent, but also by the nature of the investigated disease causing cognitive impairment and high emotional distress. The inevitable and likely substantial confounding by indication could limit the interpretability of observational research, but can be circumvented by using practice variation and statistical methods like instrumental variable analysis.

In conclusion, findings of this study will provide evidence on the effectiveness of treatment strategies in patients with subarachnoid haemorrhage caused by the rupture of an IA, hopefully contributing to future standardised high value care.

## Data Availability

Not applicable.

## Declarations

### Objection by incapacitated subjects

This procedure will be done according to the protocol “Samenwerkende Topklinische Ziekenhuizen Standard Operating Procedure Informed Consent”. If an eligible patient is identified, prospective data collection will start. If the patient is incapacitated at time of inclusion, a representative will be informed about the study and is asked to read the information letter and sign the consent form.

In the case a patient becomes capacitated during the research, an information letter and informed consent form is provided, and informed consent of the patient is gained. If the patient refused to participate in the study, all study data will be destroyed. If a patient becomes incapacitated during the study period, informed consent will be gained again from the representative. In case the representative refused to further participate, all data up to the moment the participant became incapacitated will be used for analysis.

If a patient of whom the legal representative had previously given consent because of incompetence becomes competent and refuses consent to the trial, acquisition of data will cease and data acquired up until then will be used for analysis. If a subject dies during data acquisition before formal consent was given, consent will be assumed and information will be given to legal representatives on how to actively withdraw their consent.

### Benefits and risks assessment, group relatedness

Patients will receive normal care during their hospital stay. For study purposes, health questionnaires and functional outcome will be assessed at follow-up. During follow-up, patients will be asked to complete several questionnaires to assess treatment success. These test and questionnaires will take approximately 2 h to complete.

Measurements will be done in the hospital, the outpatient clinic, the rehabilitation centre, or in the patient’s home (in the case of patient reported questionnaires). Patients may experience temporary fatigue from completing questionnaires.

### Compensation for injury

The sponsor/investigator has asked the ethical committee to waive the obligatory liability insurance as described in article 7 of the WMO. This is based on the strictly observational nature of the study and the low possibility of damage occurring caused by the study.

### Handling of data and materials

All data will be treated confidentially and coded and will be registered on data registration forms. Data management will be handled at the Department of Neurosurgery of Leiden University Medical Centre. Data will be stored in an electronic data capture and management system. Imaging data from participating centres will be stored separately in a picture archiving and communication system at the main study centre (imaging from admission and 1, 5- and 10-year follow-up). A list that contains the study participant and associated study identification code will be used to identify an individual study participant if necessary. Every study site has a specific identifier, and subjects will be assigned a study code based on an increasing number. This identification code will not be based on the participant’s initials or date of birth. The list that links the study identification code to the study participants will be kept on a designated partition on the hard drive of the research centre. This part will be protected by a password. People who have access to this partition are local researchers and quality monitor appointed by the principal investigator. These procedures comply with the European law: Algemene verordening gegevensbescherming (AVG). All raw data will be collected and stored at the main study site, including the trial master file, investigator site file, informed consent forms, study data and imaging data. The acquired data will be stored for 15 years after the study is finished and will be used for further research if the participant has provided consent for the data to be used in further studies.

### Monitoring and quality assurance

Data monitoring and quality assurance will be handled by the Department of Neurosurgery at Haaglanden Medical Centre. Study monitoring will be done on a yearly basis in each participating centre. An auditing log will be enabled of all study events, changes in study settings and study data. The informed consent forms, the investigator site file and the trial master file, as well as the study data will be monitored.

## Abbreviations

ABR: ABR form, General Assessment and Registration form, is the application form that is required for submission to the accredited Ethics Committee (In Dutch, Algemene Beoordeling en Registratie)
ADL: Activities of Daily Life
AE: Adverse Event
AR: Adverse Reaction
aSAH: Aneurysmal Subarachnoid Haemorrhage
AVG: Algemene Verordening Gegevensbescherming
AVM: Arteriovenous Malformation
BRT: Barrow Ruptured Aneurysm Trial
CA: Competent Authority
CI: Confidence interval
CCMO: Central Committee on Research Involving Human Subjects (in Dutch: Centrale Commissie Mens-gebonden Onderzoek)
CRF: Case Report Form
CSF: Cerebrospinal Fluid
CT: Computed Tomography
CV: Curriculum Vitae
dAVF: Dural Arteriovenous Fistula
DCI: Delayed Cerebral Ischemia
DSMB: Data Safety Monitoring Board
DCI: Delayed cerebral ischemia
EU: European Union
EudraCT: European drug regulatory affairs Clinical Trials
EQ-5D: Health-related quality of life measurement instrument
GCP: Good Clinical Practice
HADS: Hospital Anxiety and Depression Scale
HMC: Haaglanden Medical Centre, The Hague
IA: Intracranial Aneurysm
IB: Investigator’s Brochure
IC: Informed Consent
ISAT: International Subarachnoid Aneurysm Trial
IMP: Investigational Medicinal Product
IMPD: Investigational Medicinal Product Dossier
LUMC: Leiden University Medical Centre, Leiden
METC: Medical research ethics committee (in Dutch: Medisch Ethische Toetsing Commissie)
METC LDD: Medical Ethics Committee Leiden The Hague Delft
mRS: Modified Rankin Scale
QALY: Quality Adjusted Life years
RCT: Randomized Controlled Trial
(S)AE: (Serious) Adverse Event
SAH: Subarachnoid Haemorrhage
SPC: Summary of Product Characteristics (in Dutch: officiële productinfomatie IB1tekst)
Sponsor: The sponsor is the party that commissions the organisation or performance of the research, for example a pharmaceutical company, academic hospital, scientific organisation or investigator. A party that provides funding for a study but does not commission it is not regarded as the sponsor, but referred to as a subsidising party
SUSAR: Suspected Unexpected Serious Adverse Reaction
TICS-m: Telephone Interview for Cognitive Status
UNCH: University Neurosurgical Centre Holland Leiden – The Hague
WEB®: Woven EndoBridge®
WFNS: World Federation of Neurosurgical Societies
WMO: Medical Research Involving Human Subjects Act (in Dutch: Wet Medisch- wetenschappelijk Onderzoek met Mensen
